# Analgesia quality index improves the quality of postoperative pain management: a retrospective observational study of 14,747 patients between 2014 and 2021

**DOI:** 10.1186/s12871-023-02240-8

**Published:** 2023-08-19

**Authors:** Di Wang, Yihui Guo, Qian Yin, Hanzhong Cao, Xiaohong Chen, Hua Qian, Muhuo Ji, Jianfeng Zhang

**Affiliations:** 1 Department of Anesthesiology, the Second Affiliated Hospital of Nanjing Medical University, Nanjing, China; 2 Department of Anesthesiology, The People’s Hospital of Pizhou, Pizhou Hospital affiliated to Xuzhou Medical University, Xuzhou, Jiangsu China; 3 Department of Anesthesiology, Affiliated Hospital of Nantong University, Nantong, Jiangsu China; 4 Department of Anesthesiology, Tumor Hospital Affiliated to NanTong University, Nantong, Jiangsu China

**Keywords:** Analgesia quality index, Artificial intelligence patient-controlled analgesia, Postoperative pain management, Moderate-to-severe pain, Adverse reactions

## Abstract

**Background:**

The application of artificial intelligence patient-controlled analgesia (AI-PCA) facilitates the remote monitoring of analgesia management, the implementation of mobile ward rounds, and the automatic recording of all types of key data in the clinical setting. However, it cannot quantify the quality of postoperative analgesia management. This study aimed to establish an index (analgesia quality index (AQI)) to re-monitor and re-evaluate the system, equipment, medical staff and degree of patient matching to quantify the quality of postoperative pain management through machine learning.

**Methods:**

Utilizing the wireless analgesic pump system database of the Cancer Hospital Affiliated with Nantong University, this retrospective observational study recruited consecutive patients who underwent postoperative analgesia using AI-PCA from June 1, 2014, to August 31, 2021. All patients were grouped according to whether or not the AQI was used to guide the management of postoperative analgesia: The control group did not receive the AQI guidance for postoperative analgesia and the experimental group received the AQI guidance for postoperative analgesia. The primary outcome was the incidence of moderate-to-severe pain (numeric rating scale (NRS) score ≥ 4) and the second outcome was the incidence of total adverse reactions. Furthermore, indicators of AQI were recorded.

**Results:**

A total of 14,747 patients were included in this current study. The incidence of moderate-to-severe pain was 26.3% in the control group and 21.7% in the experimental group. The estimated ratio difference was 4.6% between the two groups (95% confidence interval [CI], 3.2% to 6.0%; *P* < 0.001). There were significant differences between groups. Otherwise, the differences in the incidence of total adverse reactions between the two groups were nonsignificant.

**Conclusions:**

Compared to the traditional management of postoperative analgesia, application of the AQI decreased the incidence of moderate-to-severe pain. Clinical application of the AQI contributes to improving the quality of postoperative analgesia management and may provide guidance for optimum pain management in the postoperative setting.

## Background

Most surgical patients face acute postoperative pain, but evidence suggests that less than half of patients receive adequate postoperative pain relief [1–3]. Effective postoperative pain management has become a major health care goal in many medical disciplines [4]. Inadequate management of postoperative pain is associated with delayed recovery time, opioid dependence, chronic pain, and additional medical expenses [5–7]. Many rewarding attempts have been made to improve the quality of postoperative pain management worldwide, such as acute pain services (APSs) [8, 9] and quality improvement (QI) programs [10]. However, multinational surveys have revealed that significant clinical challenges still exist in postoperative pain management [11–15]. Nowadays, with the rapid development of machinery and deep learning algorithms, modern intelligent postoperative pain management reflects a shift in anesthesia thinking—away from a simple focus on pain relief towards a focus on the overall quality of analgesia.

Patient-controlled analgesia (PCA) is the standard practice, yielding greater satisfaction and preference than traditional parenteral analgesia managed by anesthesiologists and nurses [16, 17]. Patient-controlled analgesia enables patients to self-administer small intravenous boluses of an analgesic at predetermined doses and frequencies [18]. Nevertheless, traditional PCA has limitations, including dispersion of PCA pump without direct or instant connection with medical personnel, lack of real-time PCA pump information, and the inability to automatically collect and analyze postoperative analgesia data. The limitations associated with traditional PCA not only impact patient safety and satisfaction but also hinder the sustainable development of postoperative analgesia management [19].

In the medical field, artificial intelligence (AI) has broad application prospects in the prevention, diagnosis and treatment of diseases and even in anesthesiology [20–24], which is expected to solve the disadvantages of traditional postoperative pain management and PCA. Artificial intelligent PCA (AI-PCA) system is an innovative analgesic system that combines traditional PCA with the Internet of Things (IoT) and AI. The system connects electronic PCA pumps and other mobile terminals with a central computer sever installed with an information control system under a wireless environment has enabled remote monitoring, intelligent alarms, intelligent analysis and assessment of the PCA equipment, and automatically recording and reserving key information, is an information system solution integrating remote monitoring, information management and high-precision infusion pump for patient-controlled analgesia. By transforming the traditional passive call mode, where patients have to rely on bedside alarms or staff-initiated interventions, into an active service, the AI-PCA system eliminates the fear and uncertainty caused by unexpected pain and delayed response. The AI-PCA significantly enhanced the dynamic management of postoperative pain, and practice showed that AI-PCA significantly reduced the incidence of moderate to severe postoperative pain as well as relevant adverse reactions, shortened the length of hospital stays, and improved patient satisfaction with postoperative pain relief compared with the traditional PCA. Overall, the integration of patient participation, real-time monitoring, electronic record-keeping, and active service in the AI-PCA system enhances the quality of postoperative analgesic management by ensuring standardized, safe, and effective pain relief while empowering patients to have more control over their own pain management process [19, 25]. Even so, it is crucial that efficient, reliable indicators that can be systematically applied and evaluated and are predictive should be developed to achieve high quality postoperative pain management [26].

With the advent of data accumulation and the increasing application of machine learning techniques, there has been a shift towards utilizing data-driven approaches to enhance the quality of analgesic management. Analgesia quality index (AQI) is a product of the development of the Ai-PCA system and has emerged as a data-driven approach to enhance the quality of postoperative analgesia management. It addresses the need for a quantitative indicator to assess the effectiveness of analgesic interventions. The components of the AQI are derived from objective data collected from the AI-PCA system database. These data include parameters such as the autonomously controlled button pressing frequency, evaluation rate, incidence of alarms, alarm processing time, drug utilization, and basic patient information. Each of these components reflects different aspects of analgesic management, such as the quality control consciousness of medical staff and analgesic technique level, precision of medical advice, and standardization of management. To calculate the AQI, the data collected from a specific area (e.g., a hospital or a region) over a defined time period (e.g., 24 h) are analyzed using machine learning techniques and algorithms. The data is processed, and a weighted intelligence score is generated. The weights assigned to each component are determined based on their relative importance in assessing the quality of analgesic management. This weighting system ensures that different aspects of analgesic management contribute appropriately to the overall AQI. By quantifying various aspects of analgesic management, the AQI allows for a more objective assessment of the effectiveness of interventions. It serves as a tool to monitor and improve the analgesic technique level, precision of medical advice, and standardization of management. The AQI enables healthcare professionals, including anesthesiologists and nurses, to identify areas of improvement in their practices and make data-driven decisions to optimize postoperative analgesia. It promotes a higher level of quality control consciousness among medical staff and helps in enhancing the overall standard of analgesic management. In summary, the AQI is a quantitative index that combines objective data and machine learning techniques to assess the quality of postoperative analgesia management. It provides a standardized measure for evaluating the effectiveness of interventions and serves as a tool for continuous improvement in the field of analgesic care.

In this study, we exploited the wireless analgesic management system database of the Cancer Hospital Affiliated to Nantong University to explore the role and effect of the AQI in postoperative analgesia management and hypothesized that the AQI would better supervise anesthesiologists and nurses to improve the quality of postoperative analgesia management.

## Methods

### AI-PCA system

The AI-PCA system combines the IoT and AI to provide advanced features and functions for pain management. AI-PCA system is composed of intelligent infusion device, disposable special liquid storage box, wireless transmission equipment, mobile ward round system, central station and information management system, which offers remote monitoring, intelligent alarm system, intelligent analysis and evaluation, data recording and storage, dynamic management of analgesia and so on. Besides, the AI-PCA system facilitates performance evaluation of healthcare providers involved in pain management. The system generates the AQI based on various objective and subjective indicators, providing a quantitative measure of the quality of analgesia delivery. This evaluation helps identify areas of improvement and promotes continuous enhancement of pain management practices.

### Data source

**Fig. 1:**
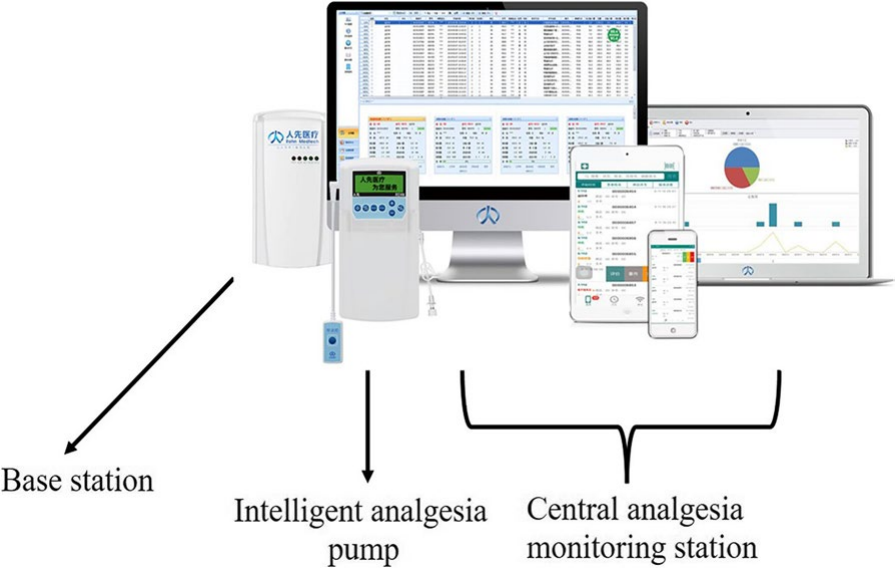
Composition of Ai-PCA system

The AI-PCA system (Jiangsu Rehn Medical Instruments Technology Co., LTD) database of the Cancer Hospital Affiliated with Nantong University was used in this retrospective study. The study was approved by the Medical Ethics Committee of our hospital, and an informed consent form was signed by the patients and their families. Created in 2014, the database gathers the usage information of all patients who have undergone postoperative analgesia using AI-PCA. Mainly composed of intelligent analgesia pump, base station (data transmission) and central analgesia monitoring station (central workstation, mobile workstation) (Fig. 1), the AI-PCA system is able to automatically capture the objective indices related to the analgesia pump (including the average pressing frequency, insufficient analgesia rate, insufficient analgesia treatment time, bubble incidence, blockage rate, drug utilization rate and so on) and subjective indicators (including the evaluation rate, poor analgesia rate, amount of basic information completed, rate of not powering off after pump withdrawal, and rate of leaving the service area, etc. Postoperative pain was measured with a numeric rating scale (NRS) and the occurrence of moderate-to-severe pain (NRS ≥ 4) and adverse reactions (nausea and vomiting, vertigo, excessive sedation, pruritus, delirium, etc.) were recorded in the Ai-PCA system every day for three days after surgery by the anesthetist and anesthesia nurses who made ward rounds.

### Study design

This was a retrospective study of postoperative analgesia involving 14,747 patients between 2014 and 2021. Despite the fact that the AI-PCA system has been in use since 2014, it was not until 2018 that an AQI was proposed and applied in the Cancer Hospital Affiliated with Nantong University. As a result, to observe the effect of the AQI in postoperative analgesia management, the patients who did not receive AQI guidance for postoperative analgesia management from June 1, 2014, to March 31, 2018, were taken as the control group (C, *n* = 7162), and the patients who received AQI guidance for postoperative analgesia management from April 1, 2018, to August 31, 2021, were taken as the experimental group (A, *n* = 7585). The AQI of group C was calculated by the original data in the wireless analgesic management system, and the AQI of group A was calculated by intelligent analysis of the AI-PCA system. The patient's AQI is included in the related medical staff's performance evaluation criteria in group A with the purpose of motivating them to continuously improve the quality of postoperative analgesia management according to the AQI.

### AQI

The AQI is a new monitoring index developed to further improve the management quality of postoperative analgesia that has obtained an Invention Patent Certificate (Patent Number: ZL 201810411669.8). It is a comprehensive index derived through real-time monitoring and assessment of the patient experience, operation status of the wireless analgesia system, work quality and efficiency of the acute pain service team. The results are calculated in percentage terms. Taking the scoring method for the insufficient analgesia rate as an example, the data of 100 hospitals in China in one month were selected for sample analysis, and a normal distribution curve is generated from these data. The mean value (μ) of analgesia insufficiency rate is 7% and the standard deviation (σ) is 6%. Thus, the score ranges from μ-σ to μ + σ, which covers approximately 68%. When the analgesia insufficiency rate is higher than μ + σ (13%), the score is 0, and when it is lower than μ-σ (1%), the score is maximal. The range from μ-σ to μ + σ is calculated in proportion (Table 1). The AQI includes 11 calculation indicators, and advice is offered following the reported scores. The calculation indicators of AQI, such as average pressing times, evaluation rate, poor analgesia rate, insufficient analgesia rate, insufficient analgesia treatment time, the rate of not turning off after pump withdrawal, the rate of leaving service area, the integrity of patient information, bubble incidence, blockage rate and drug utilization rate, are all objective data from the AI-PCA system database. By assigning appropriate weights to each indicator, the AQI combines these objective data points to generate a single score that reflects the quality of analgesic management. This score can be calculated for a specific area, such as a hospital or a certain region, over a defined time period, such as 24 h. The AQI is typically represented as a percentage-based score, ranging from 0 to 100%. A higher AQI score indicates better quality of postoperative analgesia management, while a lower score suggests areas for improvement.

**Table 1:** Introduction and score calculation method of AQI indicators

Indicators	Illustration	System configuration	Full score	Scoring formula
Basic information	Patients'name,sex,age,height,weight,operation name, ASA classification, the formula and parameters of the analgesia pump, etc Number of basic information completed = maximum number of configurations -(number of unfilled basic information/number of patients)	Maximum:9.00 Minimum:0.00	10.00	10–10 × ((9- Number of basic information completed)/9)
Average pressing times	Average pressing times = total pressing times/number of patients	Maximum:0.50 Minimum:0.00	5.00	5–5 × ((0.5- Average pressing times)/0.5)
Evaluation rate	Evaluation rate = total number of evaluations/number of patients	Maximum:1.00 Minimum:0.00	15.00	15–15 × ((1- Evaluation rate)/1)
Poor analgesia rate	Patients effectively press the automatic control button more than 4 times within 1 h Poor analgesia rate = total number of poor analgesia alarms/number of patients	Maximum:0.05 Minimum:0.00	10.00	10 × ((0.05- Poor analgesia rate)/0.05)
Insufficient analgesia rate	Invalid pressing times ≥ 3 in one lock time. Insufficient analgesia rate = total number of analgesia insufficiency alarms/number of patients	Maximum:0.13 Minimum:0.00	10.00	10 × ((0.13- Insufficient analgesia rate)/0.13)
insufficient analgesia treatment time	From the alarm of insufficient analgesia immediately to the treatment is completed and recorded in the system Insufficient analgesia treatment time = total treatment time/number of insufficient analgesia alarms	Maximum:120.00 min Minimum:30.00 min	10.00	10 × ((120- Insufficient analgesia treatment time)/90)
The rate of not power off after pump withdrawal	After pump withdrawal operation in the system, infusion is still recorded and PCA pump is still running The rate of not power off after pump withdrawal = Number of patients with infusion records after pump withdrawal/total number of patients	Maximum:0.50 Minimum:0.00	5.00	5 × ((0.5- The rate of not power off after pump withdrawal)/0.5)
The rate of leaving service area	The patient side (PCA pump) leaves the local area network or the system failure that causes an alarm of leaving the service area, so that the patient's use of PCA pump cannot be observed in real time The rate of leaving service area = number of alarms of leaving service area/number of patients	Maximum:0.50 Minimum:0.10	5.00	5 × ((0.5- The rate of leaving service area)/0.4)
Bubble incidence	Bubble incidence = total number of bubble alarms/number of patients	Maximum:0.50 Minimum:0.10	10.00	10 × ((0.5- Bubble incidence)/0.4)
Blockage rate	Blockage rate = total number of blockage alarms/number of patients	Maximum:0.50 Minimum:0.10	10.00	10 × ((0.5- Blockage rate)/0.4)
Drug utilization rate	Drug utilization rate per patient = input quantity/total solution Average drug utilization rate = total drug utilization rate/number of patients	Maximum:0.67 Minimum:0.50	10.00	10–10 × ((0.67- Average drug utilization rate)/0.17)

### Workflow and performance appraisal

**Fig. 2:**
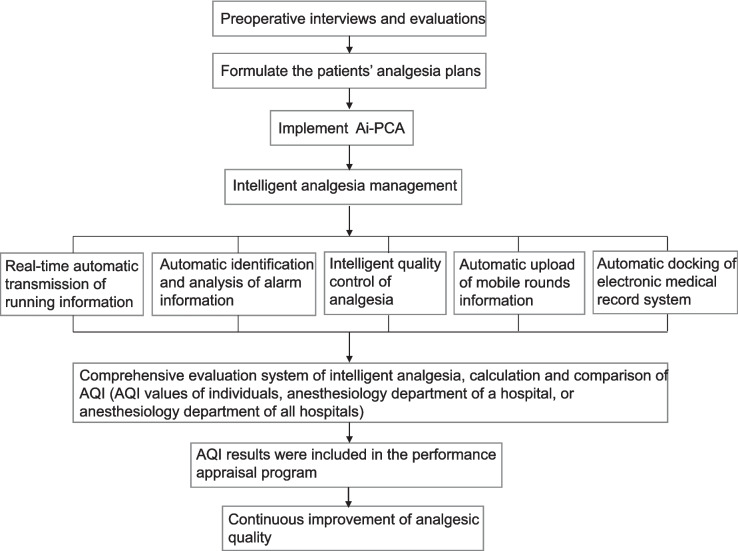
Workflow of AQI for postoperative pain management

Figure 2 is a flow chart that illustrates a work flow who received AQI guidance for postoperative analgesia. The anesthetists conducted preoperative interviews, signed the anesthesia and analgesia informed consent forms, and formulated the patients’ analgesia plans individually. The anesthesia nurses executed the anesthetists’ orders and prepared the AI-PCA pump. The AI-PCA pump was used 30 min before the end of surgery to ensure continuous postoperative analgesia and continued until 3 days after surgery. The anesthetists and anesthesia nurses made daily rounds of hospitalized patients who were using AI-PCA pumps in order to understand the analgesia effects and adverse reactions, to adjust the AI-PCA pump parameters (initial dose, single dose, continuous background infusion dose, locking time, limit dose) on demand. Postoperative pain was measured with a numeric rating scale (NRS) and the occurrence of moderate-to-severe pain (NRS ≥ 4) and adverse reactions (nausea and vomiting, vertigo, excessive sedation, pruritus, delirium, etc.) were recorded in the AI-PCA system every day for three days after surgery by the anesthetist and anesthesia nurses who made ward rounds. The real-time AQI, daily AQI, weekly AQI, monthly AQI and yearly AQI was able to be calculated; in addition, the AQI value of the individual anesthesiologist, anesthesiology department of a hospital, or anesthesiology department of all hospitals in an area at a given time could be automatically computed by the system. According to the comparison of longitudinal time and horizontal hospitals by the AQI, the postoperative analgesia management level is continuously improved by identifying and solving clinical problems. Anesthesiologists and nurses should improve their clinical practice based on these recommendations.

**Fig. 3:**
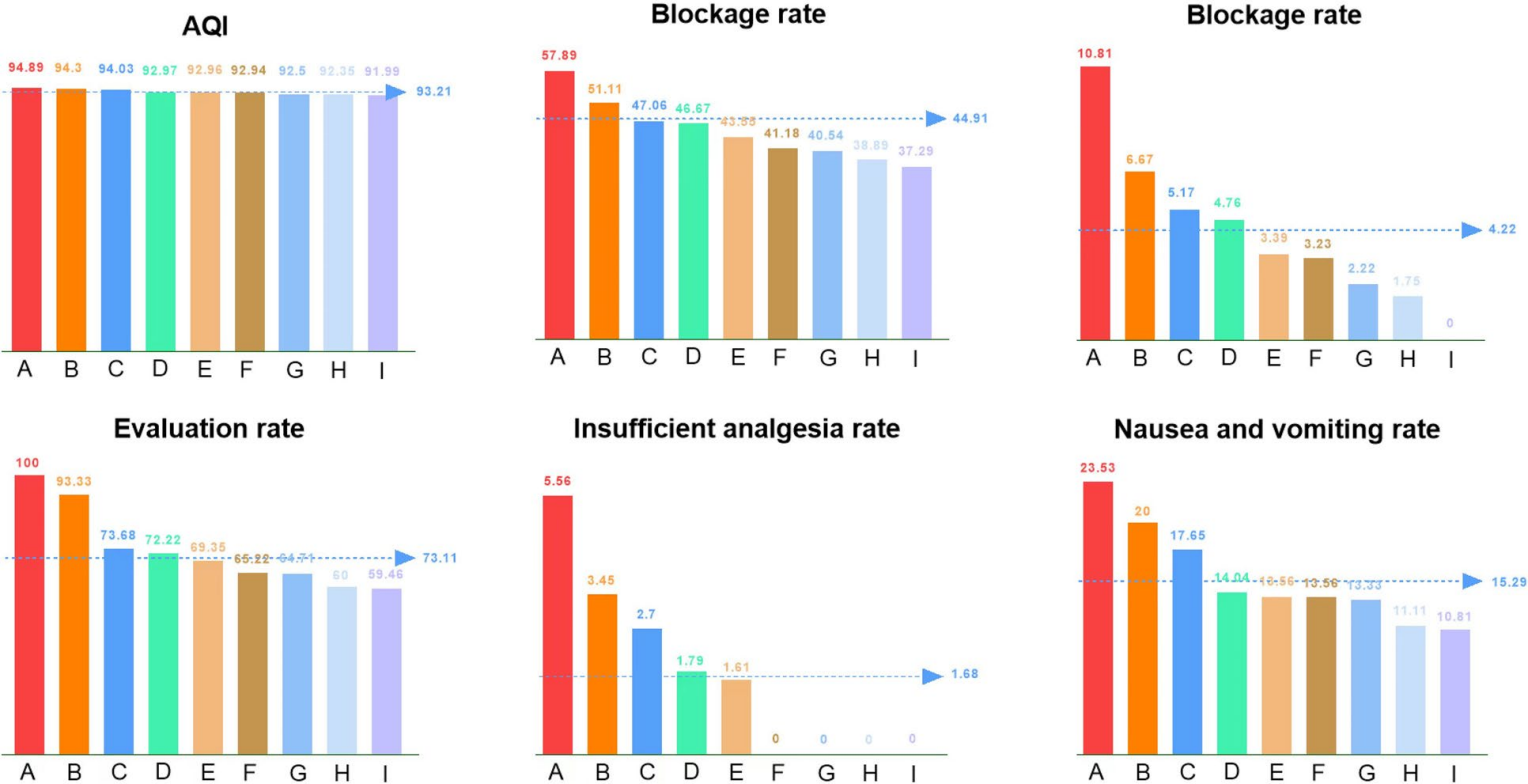
Comparison of AQI Indicators among Different Anesthesiologists

The AQI value and its indicators were compared among different anesthesiologists at a given time, and the results were included in the performance appraisal program, to urge each anesthesiologist to make continuous improvements according to their own deficiencies (Fig. 3).

### Statistical analysis

The total AQI value of the non AQI-management group was computed based on data from 7162 patients with AI-PCA postoperative pain management from June 1, 2014, to March 31, 2018. In addition, the total AQI value of the AQI-management group was calculated based on data from 7585 patients with AI-PCA postoperative pain management by the AQI from April 1, 2018, to August 31, 2021. The Kolmogorov–Smirnov test was used to evaluate whether the data meet the normal distribution. For continuous variables, the normal distribution data was represented as mean ± standard deviation (SD) and analyzed using the independent sample *t* test, and the skew data was represented as median (interquartile range, IQR) and analyzed using the Mann–Whitney U test. Categorical data which were shown as numbers and percentages were analyzed by χ2 test or Fisher's exact test.

For all analyses, unless indicated otherwise, *P* values of less than 0.05 were considered statistically significant, and two-sided statistical tests were performed. Statistical analyses were performed using either SPSS Statistical Software, version 21.0 (SPSS Inc, USA).

## Results

A total of 14,747 patients were included in this study. Table 2 shows the baseline characteristics of all participants.

**Table 2:** Baseline characteristics of participants

Characteristics	Control group (*n* = 7162)	Experimental group (*n* = 7585)
Age, mean (SD), y	60 (13)	62 (12)
Gender, n (%)		
Male	3354 (48.5)	3472 (45.9)
Female	3559 (51.5)	4099 (54.1)
Types of surgeries, n (%)		
Thoracic	2316 (32.3)	2193 (28.9)
Hepatobiliary-pancreatic	638 (8.9)	690 (9.1)
Gastrointestinal	1595 (22.3)	1921 (25.3)
Urologic	365 (5.1)	500 (6.6)
Gynecologic	1760 (24.6)	2036 (26.8)
Other	488 (6.8)	245 (3.2)

### Primary outcome

**Fig. 4:**
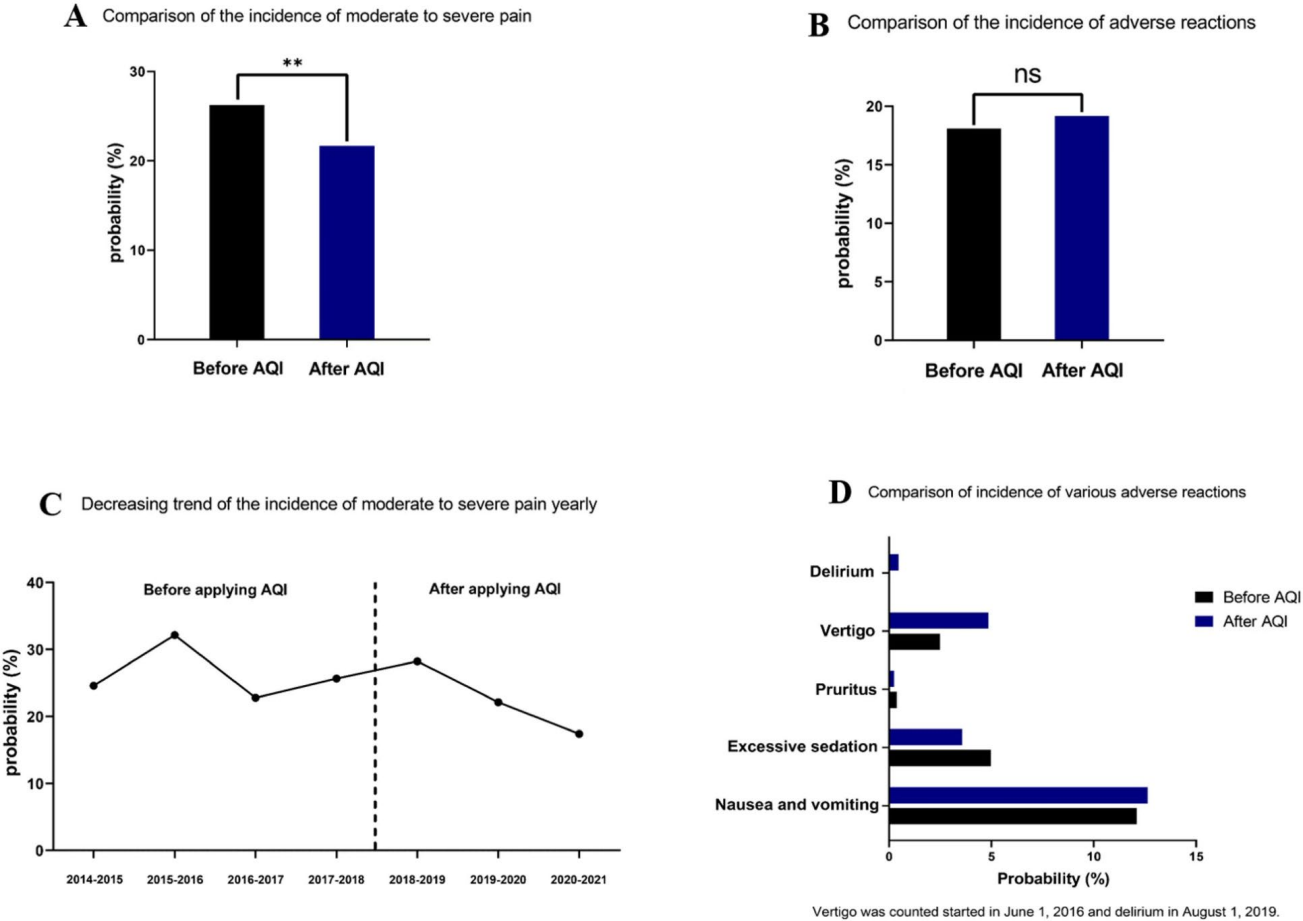
Comparison of the incidence of moderate to severe pain and adverse reactions

The incidence of moderate-to-severe pain was 26.3% in the control group and 21.7% in the experimental group. The estimated ratio difference was -4.6% between the two groups (95% confidence interval [CI], -6% to -3.2%; *P* < 0.001). There were significant differences between groups (Fig. 4A). Figure 4B shows year-to-year changes in the incidence of moderate to severe pain from 2014 to 2021.

### Second outcome

Since the incidence of vertigo and delirium began to be measured on June 1, 2016 and August 1, 2019 respectively, the total incidence of adverse events was not significantly different between the non-AQI-management group and the AQI-management group (18.1% vs. 19.2%; ratio difference, 1.1%; 95% CI, -0.2% to 2.3%; *P* = 0.09) (Fig. 4C). However, compared with the non-AQI-management group, the incidence of excessive sedation was reduced in the AQI-management group (5.0% vs. 3.6%, *P* < 0.001) (Fig. 4D).

**Fig. 5:**
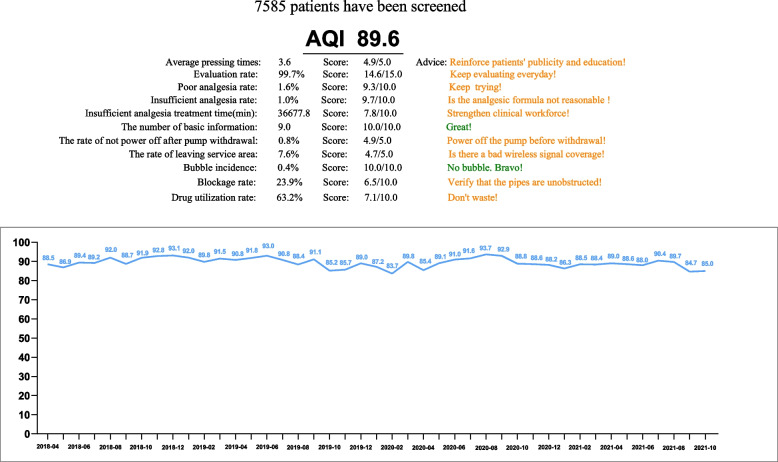
The total AQI value of the non-AQI-management group

**Fig. 6:**
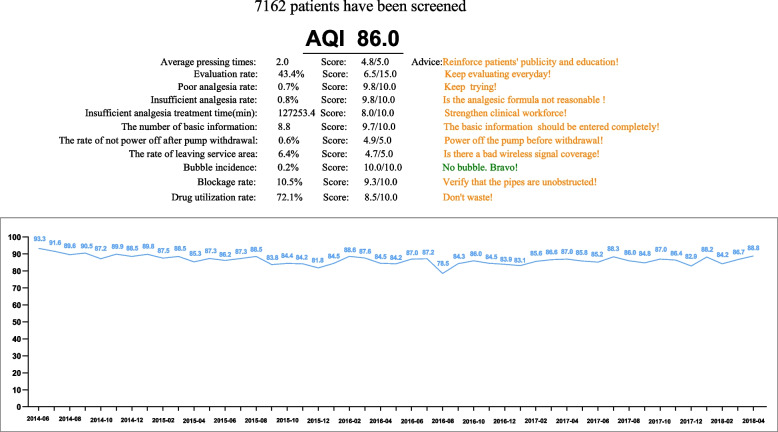
The total AQI value of the AQI-management group

**Fig. 7:**
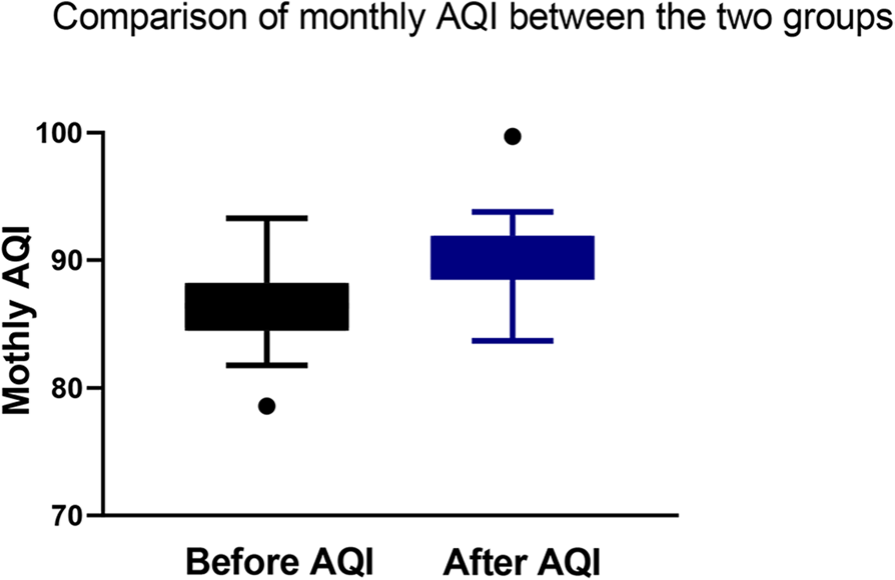
Comparison of monthly AQI between the two groups

The total AQI value of the non-AQI-management group is shown in Fig. 5, and the total AQI value of the AQI-management group is shown in Fig. 6 (86 vs. 89.6). In addition, compared with the non-AQI-management group, the monthly AQI of the AQI-management group was higher (88.5 (4.5) vs. 90.2 (3.1); median difference, 1.7; 95% CI, 0.8 to 3.1; *P* = 0.001) (Fig. 7).

After the analysis of various monthly AQI indicators in the two groups, we found that the completion of patients’ basic information was improved in the AQI-management group (8.9 (0.2) vs. 9.0 (0), *P* < 0.001). Moreover, compared with the non-AQI-management group, the evaluation rate of the AQI-management group was significantly improved (74.7 (6.8) vs. 102.2 (2.7), *P* < 0.001), the insufficient analgesia treatment time of the AQI-management group was significantly shortened (69,138.8 (122,135.2) vs. 140.5 (16,567.8), *P* = 0.019), and the rate of leaving service area was also reduced (8.5 (7.4) vs. 6.2 (5.4), *P* = 0.048). In addition, the average pressing times (3.1 (1.1) vs. 2.1 (0.8), *P* < 0.001), the insufficient analgesia rate (1.2 (0.8) vs. 0.9 (0.6), *P* < 0.001) and drug utilization rate (72.1 (5.0) vs. 64.0 (4.4), *P* < 0.001) were reduced in the AQI-management group (10.47 ± 7.23 vs. 7.23 ± 5.38) compared with those of the non-AQI-management group. However, the poor analgesia rate (0.6 (1.8) vs. 1.6 (1.1), *P* < 0.001) and the blockage rate (17.5 (4.0) vs. 24.2 (3.9), *P* < 0.001) were higher in the AQI-management group (Table 3).

**Table 3:** Indicators of monthly AQI

Indicators	Control group (*n* = 7162)	Experimental group (*n* = 7585)	*P* value
Number of basic information median (IQR)	8.9 (0.2)	9.0 (0)	< 0.001
Evaluation rate median (IQR), %	74.7 (6.8)	102.2 (2.7)	< 0.001
Insufficient analgesia treatment time median (IQR), min	69,138.8 (122,135.2)	140.5 (16,567.8)	0.019
Bubble incidence median (IQR), %	0.2 (0.6)	0.2 (0.3)	0.706
Rate of not power off after pump withdrawal median (IQR), %	0.8 (1.0)	0.6 (0.9)	0.550
Rate of leaving service area median (IQR), %	8.5 (7.4)	6.2 (5.4)	0.048
Average pressing times median (IQR)	3.1 (1.1)	2.1 (0.8)	< 0.001
Insufficient analgesia rate median (IQR), %	1.2 (0.8)	0.9 (0.6)	< 0.001
Poor analgesia rate median (IQR), %	0.6 (1.8)	1.6 (1.1)	< 0.001
Blockage rate mean (SD), %	17.5 (4.0)	24.2 (3.9)	< 0.001
Drug utilization rate mean (SD), %	72.1 (5.0)	64.0 (4.4)	< 0.001

## Discussion

In the practice of using AI-PCA to manage postoperative analgesia, the efficiency and quality of anesthetic care will inevitably affect the quality and comfort of postoperative analgesia. In this study, as an algorithm to evaluate the work quality of anesthetic doctors and nurses, the AQI may urge anesthesiologists and nurses to continuously improve their work. The main scoring items of the AQI index are related to the important process data in AI-PCA management.

### Insufficient analgesia rate and poor analgesia rate

On the one hand, the occurrence of insufficient analgesia and poor analgesia reflects a state in which the anesthesiologist does not fully evaluate the patients and does not have a solid grasp of the basic pharmacokinetics and interactions of analgesic drugs. Therefore, there are problems in the formulation and parameter setting of analgesic drugs, resulting in poor analgesic effects, which also urges anesthesiologists to absorb experience and improve drug formulations in their future work. On the other hand, the alarm caused by insufficient analgesia and poor analgesia can urge medical staff in charge of quality control in the department of anesthesiology to adjust the relevant parameters in time during the process of postoperative management (e.g., adjusting the background amount or using single doses) to alleviate the suffering of patients and improve the satisfaction and happiness of patients, which are concerns of doctors and nurses.

### Average pressing frequencies

A self-controlled pressing button is a very important part of PCA, which allows patients to administer the drug according to their own pain. Not pressing the self-control button at all may be due to a large dose of analgesic drugs or mild pain, which may increase other adverse reactions, such as excessive sedation and hypotension or even respiratory depression, which cannot be detected in time and may cause serious consequences. In addition, it is also likely that the patient does not fully understand the role of the self-control button. Therefore, the pressing frequency of the self-control button can reflect not only the accuracy of the order issued by the anesthesiologist but also whether the relevant education regarding pain and analgesic pumps was sufficient before the operation. Of course, the most important issue is that the pressing frequency is also one of the indicators of the patient's pain.

### Evaluation rate

Patient satisfaction with analgesia is an important index to measure the service quality of medical staff and is used as an outcome measure in QI studies [4, 27, 28]. Postoperative ward rounds are an important part of postoperative analgesia management. After using PCA, it is necessary daily to inquire regarding and to record the use of analgesia pumps, the degree of pain and the occurrence of adverse reactions. This scoring item of postoperative evaluation is used to evaluate how much anesthesiologists and nurses attach importance to the task and how well it is done. More importantly, it reflects the attention and care of doctors and nurses, which contributes to achieving timely feedback from patients and further improvement to enhance patient satisfaction.

### Blockage rate

Approximately 90% of the alarms are triggered by blockage of the PCA pump pipeline in the process of analgesia, which may be due to the bending of pipelines resulting from body position or other reasons, and treatment would inevitably be labor-consuming and time-costing. In addition, the nurses in the ward may clamp the analgesic pump to the patient due to adverse reactions such as postoperative nausea and vomiting and excessive sedation.

In the AI-PCA pump, a pressure valve has been developed and designed to effectively block liquid velocity caused by gravity (CN207614132U), which increases the safety of infusion. Moreover, the pressure self-checking device in the Ai-PCA pump can guarantee automatic detection and self-recovery of the pipeline after blockage (CN103007380B), which allows the patients to clamp or open the pipeline according to their own requirements and further facilitate patients’ self-regulation of analgesia. In conclusion, these devices in the AI-PCA pump improve the working efficiency of health care professionals and the quality of medical care.

At present, the incidence of pipeline blockage indicates inadequate education on the use of AI-PCA pumps, inadequate pain assessment and the presence of too much analgesic in the AI-PCA pump, resulting in patients clamping the infusion line due to adverse reactions (nausea and vomiting, dizziness and oversedation, etc.), painlessness, or altering the time of AI-PCA pump usage by clamping it to save the solution.

### Drug utilization rate

The drug utilization rate can reflect the accuracy of the ordering anesthesiologist, who should avoid blindly pursuing the analgesic effect and blindly using large doses of narcotic drugs. Such evaluation also prevents the anesthesiologist from mechanically giving every patient the same analgesic formula and analgesic pump parameters and can urge the anesthesiologist to reduce unnecessary waste and decrease the hospitalization expenses of patients.

Analgesia technology and analgesics are constantly updated and improved, but the incidence of postoperative moderate-to-severe pain still has not gone down [29, 30]. In the current study, within the past four years, the reduction in moderate-to-severe pain, the average pressing frequency and insufficient analgesia rate indicates that the effect and quality of postoperative analgesia has been improved in patients who received AQI guidance for postoperative analgesia management from April 1, 2018, to December 31, 2020. This in turn may suggest that the AQI first proposed and applied to the management of postoperative pain plays a major role. In addition, the yearly declining trend of moderate-to-severe pain indicates that the AQI calculated based on process indicators plays an increasingly important role in postoperative analgesia management, which urges doctors and nurses to strengthen the quality of medical services in order to improve postoperative analgesia and patient satisfaction. The AQI is the first attempt at postoperative analgesia management and will be perfected continuously in the future.

Although the application of the AQI in postoperative pain management reduced moderate-to-severe pain, the incidence of total adverse reactions was not significantly affected, which may be due to vertigo being noted and recorded in the system since June 2016 and delirium since August 2019. However, the incidence of over sedation was decreased, indicating that AQI may play a role in reducing the occurrence of adverse reactions. There was no significant change in the incidence of nausea and vomiting, which may be related to the increased proportion of endoscopic surgery [31, 32].

The increase in the evaluation rate and the amount of basic information as well as the reduction in the rate of leaving the service area and insufficient analgesia treatment time, reflect the improvement in clinical nursing quality, which is consistent with the change in the monthly AQI.

Since the application of a pressure valve and pressure self-test device in AI-PCA pumps in 2018, patients who feel uncomfortable can clamp the tube by themselves, and there is no need to wait for medical treatment. Therefore, the increase in the blockage rate may be due to the increase in occlusion of analgesic pump lines by the patients themselves.

With the expansion of the applied range of minimally invasive surgery (MIS) technology, the proportion of endoscopic surgery has increased (1310/7162 vs. 3202/7585), and anesthesiologists continue to administer postoperative analgesics in the traditional manner, which explains the decrease in drug utilization. On the other hand, since April 26, 2018, nonsteroidal anti-inflammatory drugs (NSAIDs) have been added to the formulation of the AI-PCA pump, while opioid analgesics have barely decreased, resulting in a decrease rather than an increase in drug utilization. Nevertheless, the reduction in the drug utilization rate leads to the wastage of analgesics and increases the chance of illegal diversion [33, 34]. The procedure-specific postoperative pain management (PROSPECT) Working Group offers recommendations for optimal pain management after different kinds of surgery with PROSPECT methodology utilizing systematic literature reviews and meta-analysis guidance [35–41]. As a result, anesthesiologists should constantly perfect the analgesic formulation of the Ai-PCA pump according to PROSPECT protocols to improve the drug utilization rate and reduce drug waste.

In conclusion, the AQI index can be used to reflect the quality of postoperative analgesia management. AI-PCA has been applied in anesthesia departments of more than 400 hospitals in China for postoperative analgesia. In this AI-PCA system, the AQI of the anesthesiology department of one hospital for a given period of time can be selected to directly reflect the quality of analgesia in this department; moreover, the AQI of different medical staffs, different departments and different hospitals can be selected for analysis and comparison.

At present, the AQI is only a preliminary exploration of postoperative pain management, but significant results have been obtained from our study. In the future, more process indicators in clinical practice will be discovered and used to calculate the AQI, forming a more comprehensive index to evaluate postoperative pain management and promote the development of analgesia.

## Conclusions

This current study revealed that compared to the traditional management of postoperative analgesia, application of the AQI decreased the incidence of moderate-to-severe pain postoperatively and improved the quality of postoperative analgesia management. Clinical application of the AQI may provide guidance for optimum pain management in the postoperative setting. The study suggests that the AQI can be used as a monitoring tool to supervise anesthesiologists and nurses, improve the quality of postoperative analgesia management, and facilitate continuous quality improvement through the Plan-Do-Check-Act (PDCA) cycle. It emphasizes the potential of machine learning and wireless technology in providing intelligent and data-driven solutions for postoperative pain management, promoting efficiency, and enhancing patient satisfaction.

## Data Availability

The datasets used and analyzed during the current study are available from the corresponding author on reasonable request.

## Abbreviations

AI-PCA: Artificial intelligence patient-controlled analgesia
AQI: Analgesia quality index
NRS: Numeric rating scale
CI: Confidence interval
APSs: Acute pain services
QI: Quality improvement
PCA: Patient-controlled analgesia
AI: Artificial intelligence
IoT: Internet of Things
AQI: Analgesia quality index
SD: Standard deviation
IQR: Interquartile range
MIS: Minimally invasive surgery technology
NSAIDs: Nonsteroidal anti-inflammatory drugs
PROSPECT: Procedure-specific postoperative pain management
PDCA: Plan-Do-Check-Act
